# Launching a new era for *Short Communications*in *Journal of Synchrotron Radiation*

**DOI:** 10.1107/S1600577526006740

**Published:** 2026-06-29

**Authors:** Dibyendu Bhattacharyya, Kristina Kvashnina, Makina Yabashi

**Affiliations:** a Atomic and Molecular Physics Division Bhabha Atomic Research Centre Mumbai400085 India; b Rossendorf Beamline, ESRF – The European Synchrotron, 71 Avenue des Martyrs, 38000Grenoble, France; c Beam Line Research and Development Group, XFEL Research and Development Division, RIKEN SPring-8 Center, 1-1-1 Kouto, Sayo-cho, Sayo-gun, Hyogo679-5148, Japan

**Keywords:** *Journal of Synchrotron Radiation*, short communications

## Abstract

*Short Communications* articles in *Journal of Synchrotron Radiation* – rapid dissemination of novel and impactful results.

*Journal of Synchrotron Radiation* is re-launching its *Short Communications* section as a way to publish interesting results rapidly. This format will provide a dynamic platform for publishing breakthrough research with exceptional speed and visibility. Designed for truly novel and high-impact findings, this format enables researchers to share their most important discoveries with the scientific community without delay, ensuring timely recognition and rapid dissemination.

In order to handle these articles quickly, the Main Editors will edit these submissions and ask Co-editors to act as reviewers. The Main Editors aim to provide an initial decision within 48 hours of receipt of the manuscript. If the manuscript is sent for peer review, reviewer reports should be returned within two weeks and authors should be contacted within two days of the Main Editor receiving the reports. We aim to publish articles online within five working days of acceptance.

Submissions should present complete, well supported findings of immediate interest, not preliminary observations, fragmented results, or data resembling a laboratory notebook. The work must be sufficiently validated to support its main claims within the concise format, and authors should indicate the potential impact on the synchrotron field and why the article should be handled rapidly.

To keep them short, the articles will have a limit of 2000–3000 words (including references) and a maximum of two tables and two figures. They should be written so as to be accessible to a broad readership and focus on a clearly defined, significant result.

The Main Editors hope this format will make *Journal of Synchrotron Radiation* an increasingly attractive platform for the rapid dissemination of novel and impactful results, providing a dedicated venue for concise publications that enable the swift communication of key scientific advances to the broader research community.

We look forward to receiving your articles.

